# Thrombin, a Mediator of Coagulation, Inflammation, and Neurotoxicity at the Neurovascular Interface: Implications for Alzheimer’s Disease

**DOI:** 10.3389/fnins.2020.00762

**Published:** 2020-07-24

**Authors:** Jaclyn Iannucci, William Renehan, Paula Grammas

**Affiliations:** ^1^The George and Anne Ryan Institute for Neuroscience, The University of Rhode Island, Kingston, RI, United States; ^2^Department of Biomedical and Pharmaceutical Sciences, College of Pharmacy, The University of Rhode Island, Kingston, RI, United States

**Keywords:** vascular, inflammation, Alzheimer’s, endothelial, coagulation, therapeutics

## Abstract

The societal burden of Alzheimer’s disease (AD) is staggering, with current estimates suggesting that 50 million people world-wide have AD. Identification of new therapeutic targets is a critical barrier to the development of disease-modifying therapies. A large body of data implicates vascular pathology and cardiovascular risk factors in the development of AD, indicating that there are likely shared pathological mediators. Inflammation plays a role in both cardiovascular disease and AD, and recent evidence has implicated elements of the coagulation system in the regulation of inflammation. In particular, the multifunctional serine protease thrombin has been found to act as a mediator of vascular dysfunction and inflammation in both the periphery and the central nervous system. In the periphery, thrombin contributes to the development of cardiovascular disease, including atherosclerosis and diabetes, by inducing endothelial dysfunction and related inflammation. In the brain, thrombin has been found to act on endothelial cells of the blood brain barrier, microglia, astrocytes, and neurons in a manner that promotes vascular dysfunction, inflammation, and neurodegeneration. Thrombin is elevated in the AD brain, and thrombin signaling has been linked to both tau and amyloid beta, pathological hallmarks of the disease. In AD mouse models, inhibiting thrombin preserves cognition and endothelial function and reduces neuroinflammation. Evidence linking atrial fibrillation with AD and dementia indicates that anticoagulant therapy may reduce the risk of dementia, with targeting thrombin shown to be particularly effective. It is time for “outside-the-box” thinking about how vascular risk factors, such as atherosclerosis and diabetes, as well as the coagulation and inflammatory pathways interact to promote increased AD risk. In this review, we present evidence that thrombin is a convergence point for AD risk factors and as such that thrombin-based therapeutics could target multiple points of AD pathology, including neurodegeneration, vascular activation, and neuroinflammation. The urgent need for disease-modifying drugs in AD demands new thinking about disease pathogenesis and an exploration of novel drug targets, we propose that thrombin inhibition is an innovative tactic in the therapeutic battle against this devastating disease.

## Introduction

The societal burden of Alzheimer’s disease (AD) is staggering, with current estimates suggesting that 5.8 million Americans and 50 million people world-wide have AD (Alzheimer’s Association, 2019). Identification of new therapeutic targets is a critical barrier to our ability to develop a disease-modifying therapy for this devastating disease. A large body of data implicates vascular pathology and cardiovascular risk factors, such as atherosclerosis and diabetes, in the development of AD. Though the mechanisms whereby these risk factors contribute to pathological processes in the AD brain have not been defined fully, it has been suggested that inflammation plays a key role in both cardiovascular disease and AD (Grammas, 2011). It is clear that a better understanding of the relationship(s) among cardiovascular risk factors, inflammation, and neurodegeneration has the potential to reveal novel therapeutic targets in the battle against AD.

Recently, a number of laboratories have provided evidence that certain elements of the coagulation cascade may initiate and/or support inflammation in the brain (Davalos and Akassoglou, 2012; De Luca et al., 2017; Göbel et al., 2018). The proinflammatory properties of the protein fibrin have attracted particular attention [reviewed in Cortes-Canteli et al. (2012), Petersen et al. (2018)]. Fibrin increases expression of a number of inflammatory and oxidative mediators, activates glial cells, and disrupts the blood-brain barrier (BBB). Another potentially important protein related to both coagulation and inflammation is thrombin. Thrombin is widely appreciated for its contribution to fibrin formation and platelet aggregation in response to vascular injury. Importantly, thrombin is also a pleiotropic enzyme that is capable of triggering a large and diverse number of cellular events through receptor-mediated activation of protease-activated receptors (PARs) (Coughlin, 2005). Levels of both thrombin and the thrombin receptor PAR-1 are elevated in AD (Sokolova and Reiser, 2008; Krenzlin et al., 2016) and thrombin expression is increased in brain microvessels collected from AD patients (Grammas et al., 2006). As we discuss below, thrombin can act in both a paracrine and autocrine manner to stimulate a noxious feed-forward cycle that likely contributes to neuroinflammation in the AD brain. The evidence summarized herein suggests that thrombin is a convergence point for AD risk factors and that thrombin-based therapeutics might target multiple points of AD pathology, including neurodegeneration, vascular activation, and neuroinflammation. We will present evidence that supports the hypothesis that thrombin could be a heretofore unexplored target for AD therapeutics.

## Coagulation and Inflammation: Functionally Linked Processes

Close integration and extensive crosstalk between coagulation and inflammation pathways are critical to the body’s response to injury (Levi and van der Poll, 2005; Esmon, 2008). Both processes utilize numerous bioactive mediators and cellular effectors that interact in a coordinated manner. Inflammatory proteins, such as cytokines, play a central role in the activation of coagulation (Levi and van der Poll, 2005). Meanwhile major coagulation factors, such as tissue factor, fibrinogen, and thrombin, are drivers of inflammation (Jennewein et al., 2011; Davalos and Akassoglou, 2012; Kalz et al., 2014; Witkowski et al., 2016; De Luca et al., 2017). Under normal physiological conditions these intertwined systems work in homeostatic balance, but dysregulation of this crosstalk likely contributes to cellular injury and disease pathogenesis.

### Effect of Cytokines on Coagulation

Cytokines, particularly interleukin (IL)-6, tumor necrosis factor-α (TNF-α), and IL-1β, stimulate procoagulant effects both directly and indirectly. These proteins initiate the extrinsic coagulation pathway through up-regulation and activation of tissue factor (Nawroth and Stern, 1986; Witkowski et al., 2016). Blocking tissue factor greatly inhibits inflammation-induced thrombosis, and inhibition of IL-6 specifically blocks tissue factor-dependent thrombin generation (Levi et al., 1997). Cytokines, especially TNF-α, can also initiate inflammation-mediated platelet activation and clumping in the blood (Page et al., 2018). Inflammatory cytokines can inhibit the anticoagulant feedback pathways, resulting in increased thrombin and fibrin production (Levi and van der Poll, 2005). Both TNF-α and IL-1β can reduce activated protein C (APC) via down-regulation of thrombomodulin, an important cofactor for APC’s anti-inflammatory and anti-coagulant activity (Nawroth and Stern, 1986).

### Coagulation Factors Drive Inflammation

Tissue factor, the main driver of the extrinsic coagulation pathway, can induce proinflammatory effects. This includes increases in the production of inflammatory cytokines, adhesion molecules, chemokines, and growth factors (Witkowski et al., 2016). These proinflammatory effects are largely mediated through tissue factor-activation of the protease thrombin (see below). Fibrin is the primary end-product of the coagulation system, but it also has proinflammatory characteristics. Fibrinogen and fibrin both have been shown to induce leukocyte migration, and directly modulate the inflammatory response of both leukocytes and endothelial cells. Fibrin induces the expression of several inflammatory cytokines and chemokines and increases the production of reactive oxygen species (ROS) (Jennewein et al., 2011). Similarly, fibrin has been shown to have extensive proinflammatory effects within the central nervous system (CNS), including activation of glia and disruption of BBB function (Petersen et al., 2018). Fibrinogen activates microglia in a CD11b-dependent manner; this activation is related to perivascular clustering, axonal degeneration, spine elimination, and cognitive impairment in animal models (Davalos et al., 2012; Merlini et al., 2019). Finally, the coagulation and immune systems are directly linked through activation of IL-1α by thrombin (Burzynski et al., 2019).

## Thrombin is a Key Mediator of Coagulation and Inflammation Via Proteolytic and Receptor-Mediated Mechanisms

The coagulation cascade consists of the intrinsic and extrinsic pathways, and thrombin is a key mediator in both (Palta et al., 2014). The extrinsic system is activated by tissue factor, which is found in the subendothelial surface and is only introduced to the blood following injury. Tissue factor in the blood complexes with factor VIIIa to initiate the cascade that will eventually lead to the formation of thrombin and the cleavage of fibrinogen to fibrin (Witkowski et al., 2016). The intrinsic system is activated by factor XII, which initiates a cascade that will lead to the production of thrombin via factors X and V (Palta et al., 2014). The activation of thrombin will also activate a positive feedback loop which will continue to drive the generation of thrombin (Palta et al., 2014). Together, these systems work to increase the amount of active thrombin in the blood, which will in turn increase the insoluble fibrin available to form a clot at the site of damage.

As noted above, thrombin is a multifunctional protease that can initiate many cellular events through action at and activation of PARs. PARs are a unique class of G-protein-coupled receptors due to their unusual tethered-ligand mechanism of activation (Coughlin, 2000). Thrombin is responsible for enzymatic cleavage of the PAR N-terminus to expose a tethered ligand that intramolecularly activates the receptor. Activation of PARs by thrombin affects a multitude of functions throughout the body, including the regulation of platelet activation, cell adhesion, cell migration, angiogenesis, and inflammation (Coughlin, 2005; Kalz et al., 2014).

### The Vasculature Is a Critical Nexus for the Proinflammatory Actions of Thrombin

Thrombin moderates gene expression through a wide array signaling processes in vascular endothelial cells (Cheranova et al., 2013) and much of thrombin’s proinflammatory activity is likely due to its numerous effects on vascular endothelial cells. Thrombin acts on endothelial cells to stimulate synthesis and release of a large number of diverse bioactive proteins. Thrombin stimulation of human umbilical vein endothelial cells (HUVECs) produces changes in gene expression related to inflammation, apoptosis, and matrix integrity (Okada et al., 2006). Thrombin-treated HUVECs exhibit increased expression of intracellular adhesion molecule-1 (ICAM-1) and vascular cell adhesion molecule-1 (VCAM-1) and a related increase in monocyte adhesion (Kaplanski et al., 1998). In human aortic endothelial cells (HAECs), thrombin and high-glucose co-treatment produces increases in the expression of NADPH oxidase (NOX), inflammatory cytokines, and altered adhesion (Wang et al., 2014). Cultured endothelial cells exposed to thrombin release von Willebrand factor, express P-selectin at the plasma membrane, and produce chemokines thought to trigger the binding of platelets and leukocytes to endothelial surface (Collins et al., 1993). Thrombin can affect vessel diameter, alter endothelial cell shape, and increase permeability of the endothelial monolayer. Endothelial cells can both release thrombin and respond to this protein via functionally active thrombin receptors (PAR-1 and PAR-3). Thrombin causes endothelial activation and enhanced expression and/or release of many proinflammatory proteins including monocyte chemoattractant protein-1 (MCP-1), ICAM-1, IL-1, IL-6, and IL-8, which in turn can injure endothelial cells leading to increased release of thrombin (Colotta et al., 1994; Marin et al., 2001; Rahman et al., 2002; Miho et al., 2005; Okada et al., 2006; Yin et al., 2010; Ishibashi et al., 2014). Thus, thrombin can act in both a paracrine and autocrine manner stimulating a noxious feed-forward cycle.

## Thrombin Contributes to the Development of Alzheimer’s Risk Factors: Atherosclerosis and Diabetes

Given thrombin’s effect on endothelial cells, it is not surprising that thrombin has been implicated in the pathogenesis of atherosclerosis. Thrombin has been widely studied for its role in the pathology of atherosclerosis, a progressive and chronic inflammatory vascular disorder (Kalz et al., 2014; Jaberi et al., 2019). A role for thrombin in the development of atherosclerosis is suggested by the observation that elevated levels of thrombin and the thrombin receptor PAR are found around atherosclerotic plaques, and that thrombin formation is correlated with disease severity in coronary atherosclerosis (Borissoff et al., 2012). Thrombin facilitates recruitment of circulating monocytes to atherosclerosis plaques by increasing expression of MCP-1, ICAM-1, and VCAM-1 (Colotta et al., 1994; Minami et al., 2004; Kalz et al., 2014). The importance of thrombin is further highlighted by the finding that thrombomodulin, which inhibits thrombin by binding to it, diminishes thrombin-induced endothelial cell dysfunction in atherosclerosis (Wei et al., 2011). Thrombin produces damage to endothelial cell barriers causing leakiness and associated leukocyte migration (Kalz et al., 2014). Thrombin inhibition has been shown to decrease ROS production, improve endothelial cell and barrier function, and attenuate atherosclerotic plaque formation (Lee et al., 2012).

Thrombin activity can influence onset, progression, and qualitative properties of atherosclerotic plaques. In atherosclerotic models, increases in thrombin have been found to be associated with increases in inflammation, angiogenesis, and cell proliferation (ten Cate, 2012; Kalz et al., 2014). Genetic reduction of thrombin in APOE-/- mice diminished atherosclerosis severity (Borissoff et al., 2013). Studies have further defined thrombin’s role in atherosclerosis by finding reduced atherosclerosis-related pathology in animal models treated with thrombin inhibitors (Wei et al., 2011; Pingel et al., 2014; Preusch et al., 2015; Palekar et al., 2016). On the other hand, genetic manipulations resulting in elevated thrombin levels produce exacerbated atherosclerosis-related pathology (Borissoff et al., 2013). These findings illustrate a causative role for thrombin in the development of atherosclerosis-related pathology and inflammation, largely through thrombin’s endothelial cell-mediated effects.

Hyperglycemia, characteristic of Type 2 diabetes mellitus (T2DM), enhances thrombin generation and promotes a hypercoagulable state and high oxidative stress (Aoki et al., 1996; Undas et al., 2008; Chapman, 2013). Elevated thrombin activity has been linked with endothelial dysfunction in diabetes, including vascular inflammation and increased ROS production in Paneni et al. (2013). One mechanism whereby thrombin promotes diabetic oxidative stress is via calcium-mediated intracellular signaling pathways that regulate the transcription factor KLF14 and PLK1 kinase pathways, resulting in increased ROS production (Hao et al., 2018). Exogenous thrombin treatment has been shown to exacerbate pathology in experimental models of diabetes. In a mouse model of diabetes, thrombin treatment of pericytes results in increased barrier permeability, decreased expression of tight junction proteins, and increased expression of inflammatory cytokines (Machida et al., 2017). Induction of diabetes by streptozotocin (STZ) in mice increases the expression of PAR-1, PAR-3, and PAR-4 in the aorta. STZ-induced diabetic mice show impairment of endothelial function, while the administration of dabigatran etexilate, a direct thrombin inhibitor, significantly attenuates this endothelial dysfunction (Rahadian et al., 2020).

Patients with T2DM show increased blood thrombin levels correlated with the patient’s level of albuminuria, an indicator of cardiovascular morbidity and mortality in patients with T2DM. This suggests that thrombin may play a role in the development of macrovascular disease (Ay et al., 2012). Thrombin-related pathways have also been implicated in diabetic microvascular injury and retinopathy. Samples taken from diabetic retinopathy patients show elevated expression of thrombin and PAR-1; similar trends are found in samples from a rat model of diabetes (Abu El-Asrar et al., 2016). Obesity promotes a chronic inflammatory and hypercoagulable state that drives cardiovascular disease and T2DM. Recent studies have suggested a link between the thrombin/fibrin (ogen) axis and obesity (Kopec et al., 2017). In a high fat animal model, treatment with the direct thrombin inhibitor dabigatran etexilate ameliorates the development of obesity and severity of associated sequelae (Kopec et al., 2014).

## Thrombin Causes Vascular Dysfunction and Promotes Inflammation in the Brain

**FIGURE 1:**
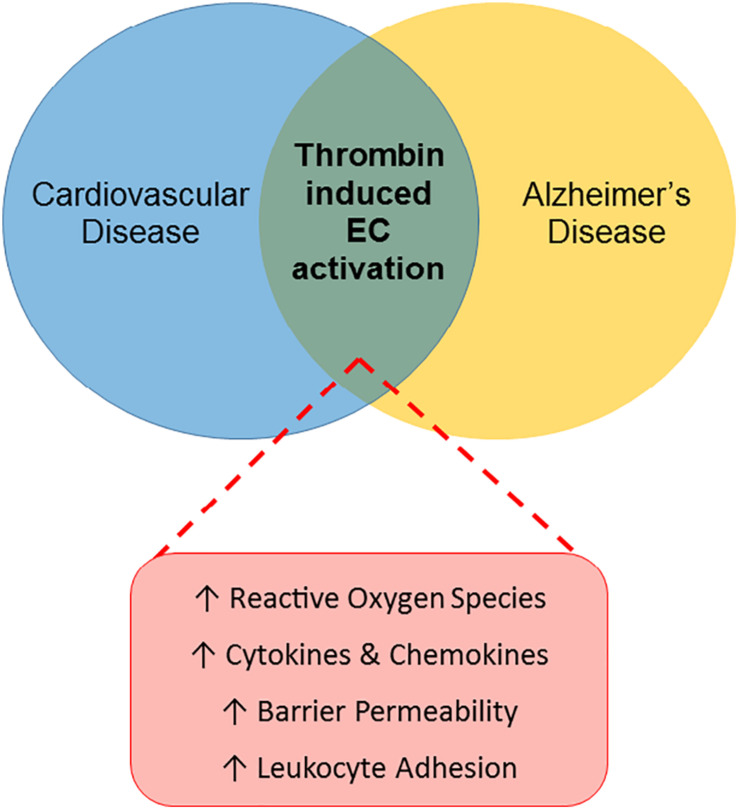
Thrombin-induced endothelial cell activation: a link between CVRFs and AD. The Venn diagram illustrates a proposed role for thrombin-mediated endothelial cell (EC) injury as a common link between cardiovascular disease and AD. Thrombin’s proinflammatory effects on ECs in the periphery, characterized by increased reactive oxygen species, cytokines and chemokines, barrier permeability and leukocyte adhesion, contribute to the pathogenesis of cardiovascular disease. Similarly, thrombin may contribute to the development of AD by causing EC activation in the cerebral microcirculation.

Largely due to its proinflammatory effects on endothelial cells, thrombin has an important role in the pathology of various peripheral vascular diseases, including atherosclerosis and diabetes, as reviewed above. Thrombin may similarly act as a pathological mediator in the CNS, through effects on the endothelial cells of the BBB (Figure 1).

### Effects of Thrombin on the BBB

Consistent with its ability to cause endothelial injury in the periphery, thrombin has been found to be involved in instances of damage and dysfunction to the brain endothelial cells of the BBB. Treatment of rat brain endothelial cells causes endothelial dysfunction characterized by increased production of ROS, nitric oxide (NO), inflammatory cytokines, and chemokines (Brailoiu et al., 2017). In human brain endothelial cells, thrombin treatment induces an inflammatory phenotype resulting in increased ICAM-1, VCAM-1, and increased mRNA expression for CXC chemokines (chemotactic for neutrophils) CXCL1 (GRO-alpha), CXCL2 (GRO-beta) CXCL8 (IL-8), and CXCL10 (IP-10) (Alabanza and Bynoe, 2012). Thrombin also increases F-actin stress fibers, disrupts tight junctions, and increases barrier permeability (Brailoiu et al., 2017). More specifically, thrombin has been found to alter barrier permeability by inducing microtubule disassembly (Birukova et al., 2004) and activating Src kinase (Liu et al., 2010). Treatment with a direct thrombin inhibitor reduces the ROS generation and expression of proinflammatory cytokines by cultured brain endothelial cells in response to hypoxia, indicating a mediating role for thrombin in the proinflammatory response of brain endothelial cells (Tripathy et al., 2013).

**FIGURE 2:**
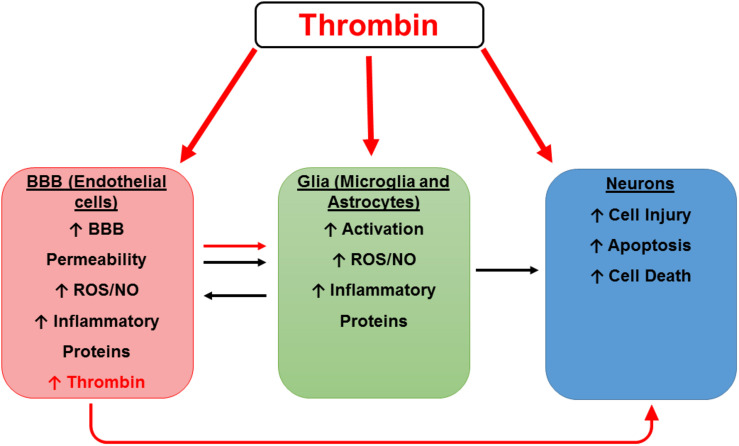
Hypothetical scheme for thrombin as a mediator of cerebrovascular activation, neuroinflammation, and neurodegeneration. A hypothetical scheme for thrombin as a mediator of cerebrovascular activation, neuroinflammation, and neurodegeneration. Thrombin can cause neuronal injury directly and indirectly by activation of endothelial cells (ECs) and glia. Thrombin can act on ECs of the blood-brain barrier (BBB) to cause endothelial activation characterized by increased permeability and an increase in reactive oxygen species (ROS), nitric oxide (NO), and inflammatory proteins including thrombin. These toxic vascular-derived products can injure neurons as well as activate neighboring glial cells to release noxious mediators which in turn cause neuronal injury.

We have previously demonstrated that thrombin message is highly expressed in microvessels from AD brains but is not detectable in control vessels (Yin et al., 2010). Similarly, Western blot analysis of microvessels shows that the thrombin protein is highly expressed in AD- but not control-derived microvessels (Grammas et al., 2006). Furthermore, injuring brain endothelial cells *in vitro* with oxidant stress (sodium nitroprusside) or an inflammatory cocktail (IL-1β, IL-6, TNF-α, lipopolysaccharide (LPS), interferon (IFN)-γ) results in thrombin release (Grammas et al., 2004). Since brain endothelial cells can both synthesize and respond to thrombin, it could function as an autocrine factor at the BBB. The importance of findings regarding the effects of thrombin on the cells of the BBB is two-fold. Directly, thrombin damage to the BBB increases permeability and may allow damaging substances, including thrombin and other inflammatory mediators from the blood, to enter the brain. Indirectly, these injured brain endothelial cells can produce their own thrombin into the brain, where it may have untoward effects on microglia, astrocytes and neurons (Figure 2).

### Neuroinflammatory Effects of Thrombin on Microglia and Astrocytes

The response of microglia to thrombin encompasses a number of processes that contribute to microglia activation and/or apoptosis. In the microglial cell line BV2, thrombin induces IL-1β release (Han et al., 2019). Thrombin has also been shown to stimulate a proinflammatory phenotype in microglia, characterized by increases in ROS, NO, and cytokine production (Lee et al., 2006; Huang et al., 2008; Yang et al., 2015; Ye et al., 2017). PAR-1 activation was found to participate in activation of microglia, indicated by up-regulation of microglial CD40 and TNF-α production (Suo et al., 2002). Thrombin, via the TNF-α/TNFR-dependent pathway, downregulates expression of the mRNA species miR-181c which in turn promotes nuclear factor (NF)-κB activity, and upregulates NF-κB target gene expression as well as increasing mixed lineage leukemia-1 (MLL1), a putative gene target for miR-181c (Yin et al., 2017). BV2 cells treated with thrombin show an increase in ROS and the nucleotide-binding domain, leucine-rich-containing family, pyrin domain-containing-3 (NLRP3) inflammasome, a component of the innate immune system, which is associated with a wide range of human CNS disorders (Ye et al., 2017).

Astrocytes show a similar shift towards a proinflammatory phenotype in response to thrombin. In rat brain astrocytes, thrombin induces matrix metalloproteinase (MMP)-9 expression and promotes cell migration via activation of the c-Src/Jak2/PDGFR/PI3K/Akt/PKCδ pathway (Lin et al., 2013). Thrombin exposure can disrupt glutamate transport in astrocytes and modulate stellation, indicating an alteration in function (Cavanaugh et al., 1990; Piao et al., 2015). Thrombin treatment *in vivo* resulted in increased glial fibrillary acidic protein (GFAP) expression in the hippocampus, indicating a proinflammatory activation in astrocytes (Mhatre et al., 2004).

## Thrombin: An Important Mediator of Neurotoxicity and Neurodegenerative Diseases

The multifunctional protease thrombin causes neuronal cell death both *in vitro* and *in vivo*, and the neurotoxic effects of thrombin are orchestrated by multiple pathways. *In vitro*, thrombin-induced neurotoxicity involves activation of PAR-1, followed by RhoA activation and cell cycle re-entry. In neurons treated with thrombin, cyclin D1 and E (early G1 cyclins) and the cyclin dependent kinase, cdk4, are activated and that these events lead to upregulation of the pro-apoptotic protein Bim and apoptosis (Rao et al., 2007). Additionally, thrombin has been demonstrated to cause a rapid influx of calcium in neurons leading to neuronal cell death (Smirnova et al., 1998). Delivery of thrombin directly into the brain by intracerebral injection causes significant neuropathology, such as enlargement of cerebral ventricles, an increased number of TUNEL-positive cells, astrogliosis, and an increase in the immunoreactivity for phosphorylated neurofilament and apolipoprotein-E (ApoE) fragments, as well as cognitive impairments including deficits in reference memory and an increase in task latency (Mhatre et al., 2004). The large body of data indicating thrombin is an important mediator of neuroinflammation and neurotoxicity support the idea that this protein is critically involved in pathological processes that contribute to the development of neurodegenerative diseases including multiple sclerosis (MS), amyolaterotrophic sclerosis (ALS), ischemia, traumatic brain injury (TBI), Parkinson’s disease (PD), and AD (Krenzlin et al., 2016).

Alterations in coagulation-related proteins have been indicated in motor-associated degenerative disorders. Both prothrombin and factor X are elevated in MS (Gobel et al., 2016). Proteomic analysis of chronic active MS lesions identified several dysregulated coagulation factors, highlighting a potential link between the coagulation cascade and MS pathology (Han et al., 2008). In experimental autoimmune encephalomyelitis (EAE), an experimental model of MS, thrombin activity precedes onset of neurological signs, increases at disease peak, and correlates with fibrin deposition, microglial activation, demyelination, axonal damage, and clinical severity (Davalos et al., 2014). The potential pathological significance of coagulation factors in neurological disease is underscored by the finding that the diminution of fibrin, the end product of thrombin’s proteolysis, either genetically or using anticoagulants, significantly reduces neurological signs, inflammation, and axonal damage in EAE (Davalos and Akassoglou, 2012). Additionally, thrombin has been linked to changes in interneuron calcium signaling and enhanced thrombospondin release in ALS (De Luca et al., 2017).

Thrombin has been extensively studied for its role in the response to ischemia (Matsuoka and Hamada, 2002). Thrombin was found to mediate neurovascular injury during ischemia (Chen et al., 2010), and increased thrombin activity was associated with subsequent neuronal damage in a model of acute focal ischemia (Chen et al., 2012). While high concentrations of thrombin seem to be neurotoxic, low concentrations of thrombin (0.01 U/mL) were found to protect against neuronal death in cellular and animal models of ischemia (Striggow et al., 2000). Increases in thrombin have also been associated with TBI. Alterations in BBB permeability have been found following TBI (Korn et al., 2005; Hay et al., 2015). In a model of TBI, BBB damage due to the injury was found to induce increases in thrombin (Piao et al., 2019).

A role for thrombin has also been identified in PD-related pathology. More specifically, thrombin-induced activation of microglia in the midbrain has been directly linked to dopaminergic neuron death (Choi et al., 2003). Thrombin receptor PAR-1 is upregulated in the brains of patients with PD (Ishida et al., 2006; Sokolova and Reiser, 2008). Recently, our lab has identified that treatment with the direct thrombin inhibitor dabigatran improved motor deficits and reduced markers of oxidative stress in a *Drosophila melanogaster* model of PD (Johnson et al., 2020). A role for thrombin in PD is further indicated by the finding that treatment with a direct thrombin inhibitor is neuroprotective in a rotenone-induced PD rodent model (Kandil et al., 2018). Finally, mice deficient in PAR-1 are protected against 1-methyl-4-phenyl-1,2,3,6-tetrahydropyridine (MPTP)-induced toxicity which causes PD-like syndrome (Hamill et al., 2007).

## A Case for Thrombin as a Driver of Neurodegeneration and Vascular Activation in AD

Thrombin has previously been suggested as a possible pathological mediator in AD (Grammas and Martinez, 2014). Thrombin is associated with typical hallmarks of AD-related pathology. Thrombin has been detected in senile plaques and in neurofibrillary tangles characteristic of this disease (Akiyama et al., 1992; Arai et al., 2006) and levels of both thrombin and the thrombin receptor PAR-1 are elevated in AD (Sokolova and Reiser, 2008; Krenzlin et al., 2016). TBI, a condition in which neurons are exposed to high thrombin levels, is associated with an increased incidence of AD (Mortimer et al., 1991; Nemetz et al., 1999). Thrombin may also contribute to ApoE-associated pathology in AD. Intracerebral administration of thrombin to rodents increases ApoE levels in the hippocampus and results in neuronal injury and cognitive deficits (Mhatre et al., 2006). The 22 kDa N-terminal thrombin-cleavage fragment of ApoE is highly neurotoxic and could contribute to ApoE-associated AD pathology (Tolar et al., 1997). Persistent thrombin signaling induces tau aggregation and related hippocampal degeneration (Suo et al., 2003; Arai et al., 2005). Thrombin induces secretion of amyloid precursor protein (APP) in endothelial cells *in vitro* (Ciallella et al., 1999) and may be involved in altered processing of APP into fragments that are found in amyloid plaques of AD brains (Igarashi et al., 1992; Chong et al., 1994; Ciallella et al., 1999). Aβ promotes thrombin generation through factor XII-mediated factor XI activation (Zamolodchikov et al., 2016). In this regard, depletion of factor XII ameliorates brain pathology and cognitive impairment in AD mice. Additionally, the factor XII-initiated contact system, of which thrombin is a key driver, is activated in AD patients and mice (Zamolodchikov et al., 2015; Chen et al., 2017).

### Vascular Activation: A Novel Pathway in AD: A Role for Thrombin

Microvessels isolated from the brains of AD patients overexpress a diverse array of neurotoxic and inflammatory proteins (Grammas and Ovase, 2001; Grammas et al., 2006). Expression of these diverse mediators is consistent with the process of vascular activation and reflects the transition of endothelial cells from a quiescent to a highly synthetic phenotype (Sumpio et al., 2002). A similar pattern of vascular activation has been identified in transgenic AD animal models (Grammas et al., 2014). Vascular activation in the AD brain has deleterious consequences for neuronal viability, as many vascular-derived factors are neurotoxic. The idea that vascular activation contributes to pathogenic events in the AD brain is strongly supported by pre-clinical studies in two AD mouse models where treatment with a vascular activation inhibitor reduced vascular-derived neuroinflammation and improved cognitive performance (Grammas et al., 2014).

Several lines of evidence support the idea that the cerebral vasculature is a convergence point for both the expression of thrombin as well as thrombin-mediated effects that contribute to neuroinflammation and neuronal injury in AD. While the majority of thrombin is produced in the liver, extrahepatic sources of locally generated thrombin, including in the brain, have been documented (Deschepper et al., 1991). Although not widely appreciated, evidence suggests the cerebrovasculature is an important source of thrombin in AD. Immunoreactivity for the major brain thrombin inhibitor, protease nexin-1, is found to be significantly decreased in AD brains, particularly around blood vessels, suggesting vascular release of thrombin (Vaughan et al., 1994). As previously stated, thrombin mRNA and protein are expressed in brain microvessels from AD patients but not detectable in brain microvessels isolated from age-matched control brains (Grammas et al., 2006; Yin et al., 2010). It is not surprising that thrombin is expressed in AD-derived brain microvessels, as *in vitro* experiments with brain endothelial cells have shown that injuring these cells causes release of thrombin (Grammas et al., 2004). Because brain endothelial cells not only produce thrombin in response to injury but respond to thrombin with a robust inflammatory response, thrombin is likely a key mediator of cerebrovascular activation in AD.

The release of vascular-derived inflammatory proteins could stimulate/activate neighboring glial cells, both microglia and astrocytes, to release inflammatory proteins as well as noxious ROS and proteases (Grammas, 2011). This noxious, neurotoxic cycle could be augmented by vascular-derived thrombin. In addition to thrombin’s inflammatory effects on brain endothelia, thrombin could contribute to deleterious and self-perpetuating neuroinflammation via induction of proinflammatory cytokines including IL-1β, IL-6, TNF-α in microglia and astrocytes (Choi et al., 2003, 2008; Lee et al., 2006; Huang et al., 2008). A recent study in the TgCRND8 AD mouse showed that long-term treatment with the thrombin inhibitor dabigatran preserved cognition, cerebral perfusion, and BBB function, and ameliorated neuroinflammation and amyloid deposition (Cortes-Canteli et al., 2019). Administration of dabigatran to transgenic AD mice diminishes ROS levels in brain and reduces cerebrovascular expression of inflammatory proteins, further supporting an important role for thrombin as a mediator of neuroinflammation in the AD brain (Tripathy et al., 2013).

## Inhibiting Thrombin: Implications for Therapeutic Intervention in AD

Increasing evidence supports a chronic procoagulant state in AD, highlighting a possible pathogenic role for thrombin in this disease (Cortes-Canteli et al., 2012). A number of studies in both human populations and animal models utilizing anticoagulant therapies support the notion that abnormalities of coagulation may promote AD pathology. Treatment of transgenic AD mice with enoxaparin, a low molecular weight heparin, reduces plaques, and Aβ accumulation (Bergamaschini et al., 2004) and improves spatial memory (Timmer et al., 2010). As previously stated, TgCRND8 AD mice on long-term dabigatran administration also showed significant improvement in AD pathology (Cortes-Canteli et al., 2019).

Earlier human studies showed improved cognition in dementia patients receiving the anticoagulant warfarin compared to untreated patients (Puccio et al., 2009). Results from these older studies in human patients hinted at a connection between dementia and coagulation abnormalities, but these data were not rigorously pursued. A more recent community-based study found that use of the thrombin inhibitor dabigatran was associated with a lower risk of new-onset dementia (Jacobs et al., 2016). An epidemiological study on atrial fibrillation (AF) patients shows increased thrombin generation and fibrin turnover in subjects with AF and dementia compared to those without dementia, and that long-term warfarin treatment appears to be protective against dementia (Barber et al., 2004; Madhavan et al., 2018).

The evidence supporting a direct connection between AF and dementia suggests the possibility that anticoagulant therapy for AF may reduce the incidence of dementia in this population, and results obtained in a number of recent population-based studies suggest that this is indeed the case (Ding et al., 2018; Field et al., 2019; Mongkhon et al., 2019; Silva et al., 2019). Interestingly, while some groups have found that all anticoagulants provide a level of protection (Friberg and Rosenqvist, 2018) most studies report that the direct oral anticoagulants (DOACs) that target thrombin are particularly efficacious (Jacobs et al., 2016; Chen et al., 2018; Cheng et al., 2018). It is tempting to conclude that DOACs reduce the risk of AF-related dementia by decreasing the incidence of thrombotic and/or embolic events, but this scenario may be overly simplistic. While there is clear evidence that AF is associated with an increased risk of ischemic stroke (Glotzer and Zieglerm, 2015) an ambitious meta-analysis of 21 studies found that the association between AF and dementia was not always dependent on the presence of clinical stroke (Kalantarian et al., 2013), raising questions regarding the mechanisms underlying this association (Chopard et al., 2018). Dietzel et al. (2018) have postulated that AF initiates and perpetuates an increase in systemic inflammation that may lead to dementia, noting recent evidence that AF is associated with increased levels of C-reactive protein (CRP), IL-2, IL-6, IL-8, TNF-α, and other inflammatory cytokines. It is probable that thrombin is a key mediator of this inflammatory state, as it is for the inflammation that accompanies AD dementia. Furthermore, the utility of DOACs in the reduction of AF-related dementia is at least partially due to the ability of these agents to inhibit thrombin’s PAR-mediated inflammatory actions.

It is therefore possible to interrupt the inflammatory cascade that contributes to AD by utilizing DOACs to inhibit thrombin. There is as yet no direct evidence that this approach will be useful in humans, but an open-label study of a hirudin (natural antithrombin anticoagulant) compound in 84 patients with mild-to-moderate AD found that hirudin plus donepezil reduced the rate of cognitive decline compared to donepezil alone, suggesting that direct thrombin inhibition may indeed be an effective strategy for treating this neurodegenerative disease (Li et al., 2012).

Although the use of any anticoagulant raises the possibility of unwanted bleeding, several studies have shown the safety and efficacy of dabigatran. Dabigatran does not interact with food, has minimal drug-drug interactions, and has a low risk of intracranial bleeding. A study documented chronic use (over 30 months) of dabigatran in patients with an average age of 71.4 ± 8.6 had minimal side effects, supporting the efficacy and safety of this drug in an elderly population (Jacobs et al., 2016). Although not without caveats, a body of data implicating thrombin in AD pathogenesis and the relative safety of dabigatran suggest a pilot clinical trial in AD patients is warranted.

## Conclusion

The urgent need for disease-modifying drugs in AD demands new thinking about disease pathogenesis and an exploration of novel drug targets. A large, and growing, literature implicates vascular pathology and vascular risk factors in the development of AD, but the specific mechanisms whereby these factors contribute to injury in the AD brain have not yet been clearly defined. It is time for “outside-the-box” thinking about how vascular risk factors, such as atherosclerosis and diabetes, as well as the coagulation and inflammatory pathways interact to promote increased AD risk. The evidence summarized here suggests that thrombin is a convergence point for AD risk factors and as such that thrombin-based therapeutics may therefore target multiple points of AD pathology, including neurodegeneration, vascular activation, and neuroinflammation. Next generation AD therapeutics should not focus on single-target drugs but rather employ a broader, combinatorial approach. We propose that thrombin inhibitors be considered as potential contributors to the dementia therapy pharmacopeia.

## Future Directions

The use of dabigatran and other DOACs as a novel strategy for the treatment of AD and possibly other neurodegenerative diseases where thrombin plays a role is warranted. In addition, with the increasing interest/use of newer tools, such as microRNAs, other innovative therapeutic approaches that target thrombin could be employed. MicroRNAs are short regulatory RNA molecules that control gene expression at the posttranscriptional level by affecting the translation and stability of mRNA targets (Simonson and Das, 2015). Evidence suggests that microRNAs are important regulators of neuronal survival, endothelial injury, and inflammatory signaling (Suárez et al., 2007; Fish et al., 2008; Harris et al., 2008; Wang et al., 2008; Wojciechowska et al., 2017; Slota and Booth, 2019). Thrombin, which is at the nexus of all three of these processes, could mediate its effects, in part, via microRNAs. The literature in this area is evolving but studies are beginning to identify thrombin-targeted microRNAs in the development of vascular injury and neuroinflammation (Table 1; Shen et al., 2011; Cowan et al., 2014; Kim et al., 2014; Mehi et al., 2014; Pan et al., 2014; Wang et al., 2014; Lin et al., 2016; Li et al., 2017; Yin et al., 2017; Barwari et al., 2018; Miao et al., 2018; Yao et al., 2019; Cui et al., 2020).

**TABLE 1:** Thrombin effects on miRNA target genes and cellular processes.

**MicroRNA (miRNA)**	**Thrombin-induced effects**	**microRNA target gene(s)**	**Cellular process**	**References**
miR-181b	↓	CARD10	Endothelial cell activation	Lin et al., 2016
miR-181c	↓	MLL1	Microglia activation	Yin et al., 2017
miR-146	↑	CARD10	Endothelial cell activation	Cowan et al., 2014
miR-146a	↓	NOX4	Inflammation	Wang et al., 2014
miR-10b	↑	HOXD10	Angiogenesis	Shen et al., 2011
miR-let7c	↑	IGF-1R	Intracerebral hemorrhage	Kim et al., 2014
miR-27b	↓	TSP-1	Angiogenesis	Miao et al., 2018
miR-21	↑	WASp	Inflammation, Fibrosis	Li et al., 2017; Barwari et al., 2018
miR-339	↑	Klotho	Inflammation	Mehi et al., 2014; Li et al., 2017
miR-223	↑	IGF-1R	Inflammation Apoptosis	Pan et al., 2014; Li et al., 2017
miR-24-1-5p	↑	PHD1	Intracerebral hemorrhage	Cui et al., 2020
miR-25-3p	↑	ADAM10	Inflammation	Yao et al., 2019

*ADAM10, a disintegrin and metalloproteinase domain-containing protein 10; CARD10, caspase recruitment domain-containing protein 10; HoxD10, homeobox D10; IGF-1R, insulin-like growth factor type 1 receptor; MLL1, mixed lineage leukemia protein-1; NOX4, NADPH oxidase 4; PHD1, prolyl hydroxylase domain 1; TSP-1, thrombospondin-1; WASp, Wiskott–Aldrich syndrome protein.*

There is increased recognition that effective therapies for AD must shift from the classical “one-size-fits-all” approach to a personalized precision medicine strategy (Hampel et al., 2017). Inter-individual variability in response to pharmacotherapy as well as molecular/physiologic “subtypes” of patients within the broad category of AD support the rationale for a personalized therapeutic approach based on unique genetic characteristics and individual lifestyle attributes (Hahn and Lee, 2019). There is growing interest in the use of pharmacogenomics to optimize the safety and efficacy of DOACs in anticoagulation therapy (Tseng et al., 2018). As more data using this approach become available, incorporating DOACs into a personalized precision medicine approach for the treatment of AD could be accomplished in the near future.

## Author Contributions

JI and PG contributed to the initial conception of the review. JI performed the literature search and wrote the first draft of the review. PG contributed to the additional literature search and assisted in writing of subsequent drafts. WR wrote an individual section of the review, prepared the figure, and assisted with editing. All authors read and approved the final version before submission.

## Conflict of Interest

The authors declare that the research was conducted in the absence of any commercial or financial relationships that could be construed as a potential conflict of interest.
